# Plants and endophytes interaction: a “secret wedlock” for sustainable biosynthesis of pharmaceutically important secondary metabolites

**DOI:** 10.1186/s12934-023-02234-8

**Published:** 2023-11-04

**Authors:** Poonam Kumari, Nikky Deepa, Prabodh Kumar Trivedi, Brajesh K. Singh, Vaibhav Srivastava, Akanksha Singh

**Affiliations:** 1 Division of Crop Production and Protection, Central Institute of Medicinal and Aromatic Plants, Lucknow, 226015 India; 2 Division of Plant Biotechnology, Central Institute of Medicinal and Aromatic Plants, Lucknow, 226015 India; 3 Hawkesbury Institute for the Environment, Western Sydney University, Penrith, NSW 2753 Australia; 4 Global Centre for Land-Based Innovation, Western Sydney University, Penrith, NSW 2751 Australia; 5 Division of Glycoscience, Department of Chemistry, School of Engineering Sciences in Chemistry, Biotechnology and Health, KTH Royal Institute of Technology, AlbaNova University Center, 106 91 Stockholm, Sweden; 6 Academy of Scientific and Innovative Research (AcSIR), Ghaziabad, India

**Keywords:** Core endomicrobiome, Endophytes, Medicinal plants, Microbiome engineering, Secondary Metabolites

## Abstract

Many plants possess immense pharmacological properties because of the presence of various therapeutic bioactive secondary metabolites that are of great importance in many pharmaceutical industries. Therefore, to strike a balance between meeting industry demands and conserving natural habitats, medicinal plants are being cultivated on a large scale. However, to enhance the yield and simultaneously manage the various pest infestations, agrochemicals are being routinely used that have a detrimental impact on the whole ecosystem, ranging from biodiversity loss to water pollution, soil degradation, nutrient imbalance and enormous health hazards to both consumers and agricultural workers. To address the challenges, biological eco-friendly alternatives are being looked upon with high hopes where endophytes pitch in as key players due to their tight association with the host plants. The intricate interplay between plants and endophytic microorganisms has emerged as a captivating subject of scientific investigation, with profound implications for the sustainable biosynthesis of pharmaceutically important secondary metabolites. This review delves into the hidden world of the "secret wedlock" between plants and endophytes, elucidating their multifaceted interactions that underpin the synthesis of bioactive compounds with medicinal significance in their plant hosts. Here, we briefly review endophytic diversity association with medicinal plants and highlight the potential role of core endomicrobiome. We also propose that successful implementation of in situ microbiome manipulation through high-end techniques can pave the way towards a more sustainable and pharmaceutically enriched future.

**Supplementary Information:**

The online version contains supplementary material available at 10.1186/s12934-023-02234-8.

## Introduction

Almost all the living organisms on Earth interact with one another in different ways, and they all coexist as a community. Similarly, plants are interconnected and impacted by the presence of microbes both above and below ground, which ultimately play a critical role in the fitness of the host plants [1]. The term "plant microbiome" broadly refers to all distinctive microbial communities that inhabit the endosphere (plant internal tissues), phyllosphere (air-plant interface), and rhizosphere (plant roots-soil interface) [2, 3]. The endophytes form tight associations with their host and thus play a pivotal role in plant growth, fitness, and development in addition to protecting against biotic and abiotic stresses via secretion of indole-3-acetic acid (IAA), siderophores, phosphate solubilizers, etc. [4–6]. In addition, some bacterial endophytes provide nitrogen to the host via nitrogen fixation [7].

In most cases, the interaction is mutually beneficial as the plant provides carbon in return for other nutrients, metabolites and protection against pests, pathogens and abiotic stresses, thereby altering the plant metabolome in various ways [8]. The bioactive secondary metabolites are derived from intermediate compounds of primary metabolism, and they are not directly required for the organism’s basic growth and development [9]. Although the precise roles of secondary metabolites in plant metabolism and physiology are not fully known, it is thought that they participate in a variety of interactions between plants and their environment, such as protection from biotic and abiotic stressors, pollinator attractants, signaling molecules etc. and thus offer a selective advantage to the sessile plants [10–13]. Additionally, once viewed as waste products, these secondary metabolites hold immense pharmaceutical importance for human health [14].

The enormous diversity of secondary metabolites in medicinal plants is gaining more recognition as a valuable reservoir of novel chemical compounds that exhibit diverse pharmacological effects. These metabolites have been isolated from higher plants in amounts close to 100,000, where medicinal plants hold a major share [15]. In the past few decades, with the advancement in science, the concept that plants individually produce metabolites has changed, and the role of microbes in modulating the metabolites has been increasingly documented [16–19]. In this regard, the ability of the endophytes to produce active compounds appears to be integral to their functions, as recent studies have demonstrated that endophytes influence the production of host secondary metabolites through a number of biochemical processes [15].

Most of the studies involving endophyte plant interactions have majorly focused on yield-enhancing attributes with less focus on how these microbes alter the plant chemistry, particularly the biosynthesis of secondary metabolites [20–22]. Nevertheless, few studies have been conducted in various non-medicinal plants, including tomato [23], rice [24, 25], soybean [26] and grapevine [27], where the impact of endophytes on secondary metabolite biosynthetic pathways have been elucidated with regard to biotic and abiotic stress tolerance mechanism. However, given that medicinal plants are highly recognised for their pharmacological attributes, a demand fueled by the pharmaceutical, herbal medicine, and nutraceutical industries, it becomes imperative to delve deeper to understand their role as it might lead to drug discovery, promote sustainable agriculture and can have wider applications in the field of medicine, environmental science, and biotechnology [28].

Thus, the focus of this article is primarily to comprehend the mechanism and role of endophytes in fine-tuning the reservoir of incredibly diverse pharmaceutically functional compounds, especially in medicinal plants. We believe that a more comprehensive understanding in this regard can be effectively utilized and harnessed for developing superior and more potent drugs derived from medicinal plants. Additionally, through this review, we have also highlighted the potential role of “core endophytes” in secondary metabolites enhancement that could be game changers in time to come. Finally, we propose that integrating multiomics approaches may help design a “customized bioformulation” of metabolites in the near future.

## Medicinal plants: gold mine of bioactive secondary metabolites

Plant metabolites are low molecular weight organic compounds classified into three main categories. This includes the primary metabolites that are usually highly conserved and directly required for the growth of plants. The second category consists of secondary metabolites comprising major groups of phenolics, terpenes, and nitrogen-containing compounds, and the third is the plant hormones that regulate organismal processes and metabolism of plants [29]. The documented plant metabolome comprises approximately over 2,000,000 characterized secondary metabolites in the plant kingdom with multidimensional applications, which majorly belong to categories like tannins, alkaloids, phenols, glycosides, volatile oils, saponins, resins, steroids, terpenoids, and bitter principles [30–32]. Among the diverse group of plants, medicinal plants are the “goldmines” of an extensive assortment of phytopharmaceutically important bioactive molecules. This is why these plants have been utilized for restoring and maintaining health, preserving and flavoring food, enhancing daily life with color and aroma, and opening doors to mystical experiences and spiritual dreams since the dawn of humanity [33]. Medicinal plants biosynthesize and accumulate a diversified class of secondary metabolites in sufficient extractable form to be economically utilized as primary resources for various commercial, scientific or technological applications [34]. Usually, the biosynthesis of primary and secondary metabolites occurs along the same pathway. The excess carbon produced during primary metabolism is essentially absorbed and stored in the form of secondary metabolites, which in time of need, are disintegrated and used again in primary metabolism, thereby maintaining the perfect balance between primary and secondary metabolic pathways in the plants [10]. Although the secondary metabolites do not take part in the plants' primary growth and development, they serve various roles in plants, from serving as defense mechanisms against pathogens, attracting pollinators and as chemical signals to other plants [33].

Bioactive molecules produced by the plant possess broad range of pharmacological and therapeutic potentialities such as antimicrobial, anticancer, antioxidant, antiviral, antitumor, anti-inflammatory, hepatoprotective, antidepressant, antidiabetic, antiatherosclerotic, antithrombotic, vasoprotective, memory enhancer, cardiovascular improver, anti-AIDS, anti-Parkinson’s disease, anti-Alzheimer’s, anti-cognitive impairment and immunoprotective effects [33, 35]. In fact, 40% of human medicine originates from natural sources, of which 25% and 13% are majorly contributed by plants and microbes, respectively [36]. For example, many plant-based novel drugs such as paclitaxel, toptecan, teniposide, ectoposide, plaunotol, vinblastin, z-guggulsterone, gomishin, nabilone, lectinan, artemisinin are being used globally for the wellbeing of the humans. The remarkable contribution of medicinal plants can be well validated by citing examples like the serpentine compound obtained from the roots of *Rauwolfia serpentina,* which is known for its anti-hypertension effect on the body. Similarly, vinblastine from *Catharanthus roseus* is applied to treat neck cancer, Hodgkins and choriocarcinoma [37]. The medicinal plants are also home to a variety of bioactive components which have an antineoplastic effect, such as vermodalin (*Vernonia amygdalina*), chebulinic acid, ellagic acid, and tannic acid (*Terminalia chebula*) and allicin (*Allium sativum*) [38].

The significant pharmaceutical contribution of several medicinal plants belonging to families like Liliaceae, Asteraceae, Rutaceae, Apocynaceae, Solanaceae, Piperaceae, Caesalpinaceae, Sapotaceae, Ranunculaceae, Apiaceae are considered potential sources of many bioactive compounds with direct application in pharmaceutical industries. It is believed that the immense biosynthetic potential of medicinal plants has not yet been fully unveiled, and it is proposed that leveraging the most recent advancements in technologies like microbiome engineering and gene editing may produce unique chemical compounds with improved or novel bioactivities.

## Diversity and distribution of endophytes in the medicinal plants

Microbial endophytes are known to be associated with all plant species, ranging from perennial trees and medicinal plants to various other crops [39, 40]. Endophytes inhabit various plant parts, including roots, leaves, stems, flowers, and seeds. The composition, richness, and population of endophytic microbial communities differ based on factors such as plant species, the specific plant compartments (e.g. roots, stems and leaves), plant age, and surrounding environmental conditions [1, 41, 42]. Evidence suggests that endophytes are acquired vertically (via seed) and horizontally from the plants soils where plant grow. Most of these endophytes are acquired from the soil by active filtration and mutual recognition between plant hosts and microbes. Plants secrete chemoattractants in various forms through root exudates to attract specific microbes. Only those microbes that can recognize those plant chemoattractants can migrate to the plant tissues, recognise the host, and penetrate the plant surface. The majority of endophytes colonize the intercellular spaces; however, many times, they are also found to be colonizing the intracellular spaces and vascular system of the plants [43].

Additionally, only endophytes with specific traits like the presence of plant polymers breakdown machinery, protein secretion system, redox response regulatory systems, quorum sensing, etc., successfully colonize and inhabit the plant endosphere, which is a relatively small space [44]. Furthermore, the interaction between the plant immune system and microbial recognition significantly impacts the successful colonization of plant tissues by microbes [1]. Continuous and progressive filtering means endophyte diversity declines from root to leaf to flower and seeds. Some microbes are shared between different compartments, while others are specialised for a particular compartment/niche (e.g. leaves). A greater understanding of the microbial makeup and abundance is now attainable because of the advancements in sequencing technology, which have also made it possible to decode the whole endophytic variety of organisms living inside the plant tissues. Compared to the high throughput next-generation sequencing technologies, traditional culture-dependent methodologies offer far less information on the variety of microorganisms. However, the direct examination of microbial activity and its interactions with the host is an additional benefit of conventional techniques [45]. The information available through modern sequencing and traditional technologies reveals the presence of both prokaryotic and eukaryotic groups of microorganisms in the plant system [4]. Amongst the prokaryotic group, most of the endophytic bacteria are distributed among four bacterial phyla, namely Proteobacteria, Firmicutes, Actinobacteria and Bacteroidetes, with major dominant genus as *Pseudomonas, Streptomyces, Bacillus, Serratia, Micrococcus, Burkholderia, Enterobacter, Mycobacterium, Rhizobium* etc*.* [4, 19, 46].

Similarly, endophytic fungi too are found to be active colonizers of the plant tissues, with Ascomycota predominating over 95% of the endospheric population of the plants, followed by Basidiomycotas [47, 48]. The dominance of ascomycetous fungi such as *Penicillium, Curvularia, Cladosporium, Aspergillus, Colletotrichum, Trichoderma,* etc., has been documented in the plant endosphere by various research groups [48, 49]. The detailed list of endophytic diversity associated with the various plant parts of medicinal plants with their respective plant growth-promoting activity has been mentioned [19, 50–107] in Additional file 1: Table S1.

## Rooted bonds: unravelling the early colonization events in plant-endophyte interaction

The exact mechanisms responsible for the relationship between plants and endophytes are still poorly understood. However, studies to date suggest that endophytic microbes, like any other microbe, share characteristic features such as rhizosphere competence, motility trait, protein-enzyme secretion systems and an ability to overcome plant immunity [108]. Due to selective pressure, the colonization of endophytic plant partners is organ and tissue-specific, ranging from roots to shoots, shoots to flowers, flowers to fruits and finally, many times to the seeds [109, 110]. According to Kandel et al. [43], successful colonization involves the deployment of endophytes by the host plant near its vicinity, followed by the attachment of the endophytes to the host plant's surface and, finally, their entry into the plants [43].

The primary step of colonization begins with the recruitment of endophytes towards the roots of the host plant with subsequent migration to stems and leaves through the xylem vessels, a process influenced by the rich exudates released by plants into the rhizosphere [111, 112]. Researchers have previously demonstrated that bacterial endophytes possess chemotactic abilities, enabling them to swim toward the plant's root system by detecting root-secreted molecules [109, 113]. A study carried out in support of bacterial chemotaxis revealed maximum utilization of the root exudates by endophytic *Stenotrophomonas maltophila* RCT31, which ultimately resulted in enhanced plant growth of a medicinal legume *Clitoria ternatea* L. [114]. Likewise, fumaric acid released by *Panax notoginseng* significantly stimulated the chemotaxis ability, growth and biocontrol-related genes of an endophytic *Bacillus amyloliquefaciens* subsp. *plantarum* YP1 [115].

**Fig. 1:**
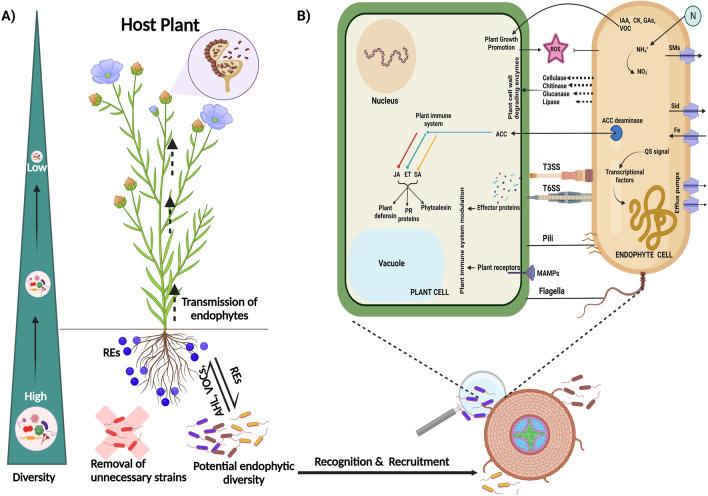
Schematic illustration depicting routes adopted by endophytes for successful colonization in the host plant. **A** Recruitment mechanism of potential endophytes and its resultant diversity gradient in the host plant. **B** Crosstalk between host plant and endophytes leads to host plant nutrient and defence management. root exudates (REs), N-Acyl homoserine lactone (AHL), volatile organic compounds (VOCs), siderophore (Sid), secondary metabolites (SMs), indole-3-acetic acid (IAA), cytokinin (CK), gibberellic acids (GAs), reactive oxygen species (ROS), 1-aminocyclopropane-1-carboxylic acid (ACC), jasmonic acid (JA), ethylene (ET), salicylic acid (SA), pathogenesis-related (PR), type III secretion system (T3SS), type IV secretion system (T6SS), microbe-associated molecular patterns (MAMPs), quorum sensing (QS)

Upon reaching the plant, bacterial endophytes employ various structural components like pili, flagella, and fimbriae, along with secretory products such as lipopolysaccharide (LPS) and exopolysaccharides (EPS), for adhering to the surface. These microbial appendages serve as propellers, translocating bacteria toward the plant's surface and overcoming repulsive obstacles [116]. In contrast, the recognition and attachment of specific hosts by fungal endophytes involve the release of fluids facilitating germling assemblage necessary for penetration. Transcriptional studies have revealed that fungal endophytes like *Piriformospora indica* secrete small secreted protein (SSP) effectors and disrupt phytohormone homeostasis during early symbiosis [117]. Upon successful attachment to the plant surface, endophytes find entry points through various plant structures, such as elongation zones, root hairs, cotyledons, stems, leaves, and flowers [118]. Passive penetration occurs through cracks, root tips, lenticels, stomata, and hydathode openings [119], while active entry is facilitated by molecules like EPS, LPS, quorum sensing signals, and chitin [21]. To avoid triggering plant resistance, endophytic microbes produce fewer cell wall-degrading enzymes and maintain lower cell densities to avoid getting detected as pathogens [120, 121]. Endophytes further utilize the xylem vessels for upward translocation, capitalizing on the low nutrient concentration in these vessels. Studies on *Paraburkholderia phytofirmans* PsJN revealed entry through the root exodermis into cortical cells via the endodermal layer [122], while the active role of alcohol dehydrogenases was observed in *Azoarcus s*p. BH72 colonization in waterlogged rice [123]. Likewise, dark septate endophytes (DSE) have been observed in fungal interactions that form alliances with plants through the root cortex, similar to arbuscular mycorrhizal fungi, employing strategies to maintain symbiosis [124]. Similarly, a unique strategy was observed in *Piriformospora indica* that secreted extracellular hydrolyzing enzyme adenosine 5'-triphosphate (eATP) in the host plant’s apoplast during later stages of interaction [125]. These findings illustrate the diverse and intricate mechanisms endophytes employ to colonize plants, shedding light on the complexity of these interactions. A diagrammatic illustration representing the early colonization events happening at plant endophyte interaction interphase has been represented in Fig. 1

## In planta secondary metabolite enhancement by the endophytes in medicinal plants

**Fig. 2:**
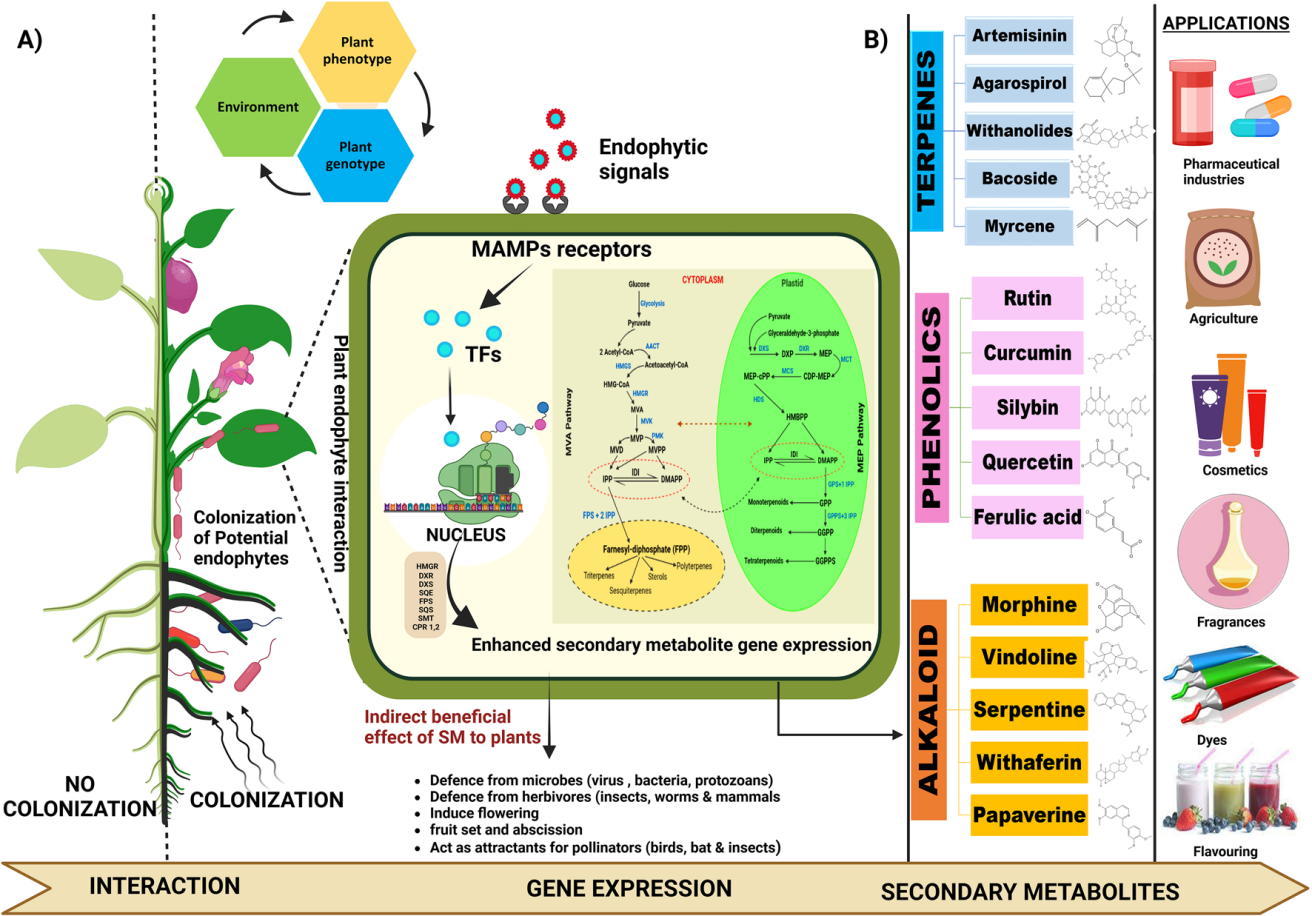
Endophyte mediated enhanced production of secondary metabolites and its applications. **A** Depicts plants inoculated with beneficial endophytes, significantly improving plant growth and development over non-inoculated plants. The zoomed-out leaf portion represents the modulation of host plant secondary metabolic pathways by multiple transcription factors regulated by upstream signals in response to endophytic colonization. **B** Different categories of bioactive secondary metabolites (structures given alongside) and their multifaceted industrial applications. microbe-associated molecular patterns (MAMPs), transcription factors (TFs), secondary metabolite (SM), mevalonic acid (MVA), methylerythritol phosphate (MEP), acetoacetyl CoA thiolase (AACT), 3-hydroxy-3-methylglutaryl-CoA synthase (HMGS), 3-hydroxy-3-methylglutaryl-CoA reductase (HMGR), mevalonate kinase (MVK), phosphor mevalonate kinase (PMK), -hydroxy-3-methylglutary-CoA (HMG-CoA), 3 mevalonate5-diphosphate decarboxylase (MVD), isopentenyl diphosphate (IPP), dimethylallyl diphosphate (DMAPP), IPP isomerase (IDI), mevalonate pyrophosphate (MVPP), farnesyl-diphosphate synthase (FPS), famesyldiphosphate (FPP), geranyl phosphate synthase (GPS), geranyldiphosphate (GPP), geranyl diphosphate synthase (GPPS), geranyl geranyl diphosphate (GGPP), geranyl geranyl diphosphate synthase (GGPPS), 1-deoxy-D-xylulose5-phosphate synthase (DXS), 1-deoxy-D-xylulose5-phosphate (DXP), 1-deoxy-D-xylulose5-phosphate reductoisomerase (DXR), MEP cytidylyltransferase (MCT), 1-hydroxy-2-methyl-2-(E)-butenyl4-diphosphate synthase (HDS), MEP-cPP synthase (MCS), squalene synthase (SQS), cytochrome P450 reductase (CPR), sterol methyltransferase (SMT), squalene epoxidase (SQE)

Recent studies reveal the enormous impact of the plant microbiome, including epiphytic and endophytic microbiomes, on the plant's overall health and performance [126]. Plant microbiomes are dynamic and can rapidly adapt to biotic, abiotic, and other environmental pressures [127, 128]. This can help host plants produce bioactive compounds at a steady rate. In addition, endophytes can mediate the re-configuration of the plant metabolome that depends on the combination of plant species/cultivar and other interacting microbial partners. Increasing evidence suggests that the interactions between the plant and the endophytic microorganisms increase the production of secondary metabolites such as alkaloids, flavonoids, and terpenoids in medicinal plants [129–133] (Fig. 2). These interactions in most cases are mutually beneficial, and a number of medicinal plants produce bioactive secondary metabolites that have significant direct and indirect impacts on the population and physiological processes of endophytic microbiota [134]. In addition, numerous research studies have indicated that root exudates play a significant role in shaping the rhizomicrobiome, affecting root-microbe interactions and endophytic diversity [135, 136]. In one study, the fungal communities associated with grapevines were influenced by the age of the leaves, with younger leaves exhibiting higher endophytic fungal diversity and richness compared to mature leaves [137]. However, the processes by which endophytes encourage the production of secondary metabolites in plants have been the subject of several investigations without a common consensus. In one such investigation, endophytes of *Panax ginseng* were found to convert ginsenosides into different forms that ultimately influenced the efficacy of the host plant [138]. It is postulated that endophytes' secondary metabolite enhancement *in planta* is linked with plants' encouragement to accumulate more photosynthetic material. This promotes the upregulation of the genes involved in the plant secondary metabolite biosynthesis pathway, changes in the genetic makeup of the plants or distinctive metabolites produced by the endophytes impacting the plant biosynthetic pathways [139].

Microbes also produce many metabolites for various purposes, including interacting with the host plant [140]. Since many metabolites are produced by microbes or by their interaction with the host plant, the focus is currently being shifted from the medicinal plants' bioactive secondary metabolites individually to linking the secondary metabolome of plants with the plant’s endomicrobiome [141]. This is because endophytic microbes are important in forming bioactive secondary metabolites such as steroids, alkaloids, polyketones, peptides, flavonoids, terpenoids, and phenols [142, 143]. Similarly, some fungi, like *Curvularia* sp. and *Choanephora infundibulifera,* are known to increase the level of vindoline, a terpenoid indole alkaloid (TIA), in the leaves of endophyte-free *Catharanthus roseus* plants by 403% and 229%, respectively. The research conducted by Pandey et al. [144] yielded molecular evidence indicating an increase in the expression of genes responsible for both the structural and regulatory aspects of the TIA biosynthesis pathways in plants inoculated with endophytes [144]. Likewise, tanshionone biosynthesis was significantly increased in hairy root cultures upon application of the polysaccharide portion of an endophytic *Trichoderma atroviride* [145]. Similarly, an integrated study involving comparative metabolomics and transcriptomics of *Epichloe festucae*-infected and uninfected ryegrass plants reported the host metabolism reprogramming in favor of secondary metabolism over primary metabolism [146].

Many studies have reported endophytes-mediated modulation of plant metabolites in recent years. For example, an endophyte-mediated accumulation of forskolin content in *Coleus forskholli* was observed by the application of three indigenous endophytes of *C. forskohlii* namely *Fusarium redolens* (RF1), *Phialemoniopsis cornearis* (SF1) and, *Macrophomina pseudophaseolina* (SF2) [147]. The findings were validated by the expression study of five key forskolin biosynthetic pathway genes, namely *CfTPS2*, *CfTPS3*, *CfTPS4*, *CfCYP76AH15*, and *CfACT1-8* [148]. Similarly, bacterial and fungal endophytes linked with the Agarwood tree (*Aquilaria malaccensis*) increased the production of agarospirol, a highly sought-after product in the pharmaceutical and fragrance industries [149]. Interestingly, tissue-specific localization of the endophytes was found to be linked with *Papaver somniferum* overall plant performance, as most leaf endophytes influenced plant development and productivity, while capsule endophytes played a key role in influencing alkaloid production [72]. Many other endophytic bacteria, such as *Pseudomonas fluorescens, Azospirillum brasilense*, *Bacillus subtilis*, *Paenibacillus polymyxa*, *Stenotrophomonas* and others, have also been reported to increase the production and accumulation of significant secondary metabolites in the host [11, 12, 16–19, 39, 129, 147, 149, 150–193] (Additional file 1: Table S2). We argue that we have only scratched the surface, and many secondary metabolites produced by plant-microbial interaction remain to be discovered and characterized. Theoretically, endophytes can impact plant metabolites' types, quantity and quality. We hypothesize that the endophytes can encourage plants for more primary and secondary metabolites through the secretion of microbial metabolites or other signal molecules, phytohormones, upregulation of defence pathways, epigenetics, etc. Additionally, they can also influence the quality of these metabolites by microbial degradation or conjugation between plant and microbial metabolites. At this point, the role of endophytic enzymes cannot be ignored, which might catalyze the initial steps of secondary metabolite biosynthetic pathways in the host plant by initiating, activating or inhibiting certain biosynthetic routes. There is also a high possibility that the endophytic microorganisms can harbor novel enzymes not present in the host plant, which may introduce new chemical reactions or biosynthetic pathways, leading to the synthesis of previously unknown secondary metabolites in the plant.

## Can core endomicrobiome be a key player in the modulation of secondary metabolites biosynthesis in medicinal plants?

Broadly, the set of operational taxonomic units consistently connected and shared by microbial communities from various but related hosts that provide specific host functions is often referred to as the “core microbiome” (CM) [194, 195]. Core microbiomes of various plants have been identified and reported [196–198]. Recently, the concept of identifying functionally important microorganisms that consistently associate with a host species is being added to the prevailing concept of CM, which is majorly based on the idea that a taxon's persistence across the spatial and temporal boundaries of a particular habitat is directly reflective of its functional importance within the niche it occupies [199, 200]. The endosphere of the plants is reported to be colonized by the community of microbial taxa with desired functional attributes, which collectively form the “core endomicrobiome” (CEM). Some genera, like *Bacillus* and *Pseudomonas,* are ubiquitous and can be found across the entire plant system [46, 201]. Previous studies have highlighted the seeds' vertical transmission of such essential microbiota over generations [202, 203]. CEMs, because of their constancy and uniformity, are thought to be essential for host fitness and hence have the ability to modulate plant microbiomes to achieve desired outcomes [204, 205]. These CEMs could be mutualists, commensalist, or occasional antagonists who, apart from directly influencing the secondary metabolite biosynthesis, might also act as key modulators for the secondary metabolite level [206]. The composition and role of CEMs have been obtained for various plants such as *Arabidopsis*, tomato, and citrus. However, limited research has been conducted to establish connections between the potential beneficial traits of the CEMs and their functions. For example, the CEMs analysis of *Oryza sativa* leaves divulged that *Acinetobacter, Enterobacter, Pseudomonas, Pantoea, Sphingomonas, Stenotrophomonas,* and *Rhizobium *genera were found to be a part of the core microbiome [207]. Likewise, Bulgarelli et al. [208] and Lundberg et al. [209] defined the microbial community structure and core endophytic population of the *Arabidopsis* root microbiome, which showed the dominance of Actinobacteria followed by Proteobacteria, Bacteroidetes and Firmicutes [208, 209]. Similar outcomes were also observed in the core-endophytic community analysis of tomato and sugarcane roots [210–213].

Since it is evident that medicinal plant endophytic microbial assemblage influences the synthesis of secondary bioactive metabolites directly or indirectly by regulating the plant metabolic pathways [214–216], the role of CEMs in this context needs further exploration. In one of the earliest studies carried out on deciphering the CEM of medicinal plants, Wicaksono et al. [217] provided valuable insights into the fact that distinct plant organs of *Leptospermum scoparium* are capable of co-harboring specific core microbial taxa that affect the bioactive properties of the plant [217]. Likewise, some signature key core microbes were also identified from two medicinal plants, namely *Achillea millefolium* L. and *Hamamelis virginiana* that were hypothesized to be the key modulators of the metabolome of the plants [48].

In another investigation, *Echinacea purpurea* has been proposed to be an excellent model herb for investigating microbiome-secondary metabolites interactions as endophytes of this plant modified the production of volatile compounds such as phenylpropanoid and alkamides [218]. Similarly, significant induction of enantiomeric naphthoquinones such as alkannin and shikonin by endomicrobiome in *Alkanna tinctoria* [16] and 19-tetrahydrocannabinol and cannabidiol in *Cannabis sativa* and morphine production in *Papaver somniferum*, were observed [215, 219]. Recently, in one of the studies, seed-associated CEMs were proposed to influence tanshinone production [220]. This postulation is based on the evidence that the identified CEMs comprise a gene reservoir associated with the terpenoid backbone synthesis, offering extra metabolic capabilities to *Salvia miltiorrhiza.* Working on the above line, our group has identified a set of core endomicrobiome genera, namely *Pseudomonas, Paenibacillus*, and *Bacillus* from *Andrographis paniculata* that, along with the introduced endophytic strain were speculated to contribute to the enhanced andrographolide content within the plant tissues [221].

Growing evidence suggests that these CEMs, in combination, probably enhance the plant’s physicochemical homeostasis by triggering secondary metabolite biosynthesis. However, there are still several gaps and challenges in this study area, as identifying effective endophytes for specific metabolite enhancement is difficult due to their vast diversity. Additionally, the ability of endophytes to enhance secondary metabolites can be species-specific and variable. Therefore, understanding the factors contributing to this specificity and variability is essential for reliable application. Complex host-endophyte interactions and the impact of environmental conditions also require further investigation for reliable applications. Despite observing shifts in endophyte diversity in response to plant metabolites, the functional consequences of these changes remain inadequately understood. Therefore, advanced genomic tools and a comprehensive understanding of endophyte biology and plant secondary metabolism are required to address these gaps and challenges.

## Bridging the gap through microbiome engineering

Irrespective of the important roles played by the microbiome, the vast bulk of the transformative potential of the microbial world is yet to be discovered and applied. It is postulated that targeted manipulation of plant microbiome via inoculation with beneficial microbes and pre- and postbiotics that promote the production of secondary metabolites can potentially help produce industrial and economically viable quantities of target metabolites. However, such approaches have their own set of limitations and have been discussed in detail in agricultural contexts [139, 222]. Recently, approaches to overcome such limitations were proposed, including explicit consideration of theoretical framework in inoculant testing and in situ microbiome manipulations/engineering [42, 222]. In this regard, modulating the plant holobiont through microbiome engineering is an emerging biotechnological approach for increasing crop yields, resilience and secondary metabolite biosynthesis in plants [223, 224].

**Fig. 3:**
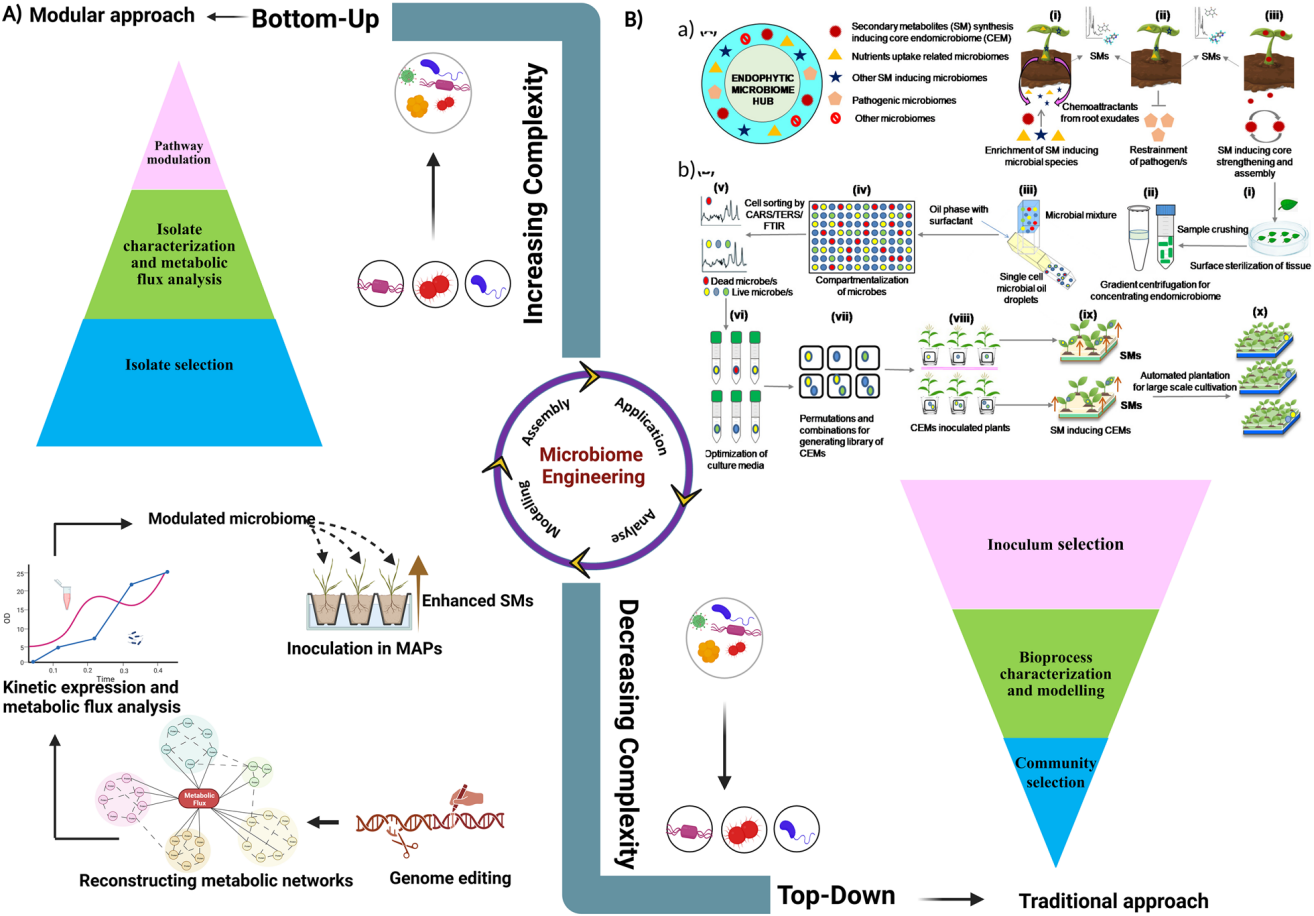
Top-down and bottom-up approaches to engineer microbiomes. **A** The panel represents the bottom-up design methodology that begins with isolates. Genome editing boosts system functions by recognizing gene editing sites that reroute metabolic flux to the intended secondary metabolites. **B** The panel represents the top-down design methodology. **a** Depiction of native endophytic micriobiome hub with interacting partners. Categories of the hub endomicrobiome are exclusively based on network topology information within a microbial network. The possible roles of secondary metabolites (SM) inducing core endomicrobiome (CEM) that are expected to mediate interactions between plants and native microbial species includes (i) Scoring of CEMs based on enrichment of SM inducing microbial species in the rhizosphere through chemoattractants released in the form of root exudates. (ii) Restrainment of deleterious biotic stressors. (iii) Bolstering of SM inducing functional CEMs by applying facilitative microbiomes. **b** Preparation of endosymbiotic cells derived from plant samples. (i) Surface sterilization of plant tissue for removal of epiphytic microbes. (ii) Sample crushing followed by gradient centrifugation for concentrating endomicrobiome. (iii) Isolation of single cell microbial droplets with CEMs using microdroplet devices (iv) Compartmentalization of single cell CEMs using microfluidic devices to separate cells from microbial mixtures. (v) Cell sorting using vibrational spectroscopy techniques like surface enhanced Raman scattering (SERS)/tip enhanced Raman scattering (TERS) and Fourier transform infrared (FTIR) (vi) Optimization of culture media for isolating CEM/s with varying nutritional requirements and growth conditions. (vii) Library development of all possible CEMs using permutations and combinations (viii) High throughput inoculation of sets of CEMs in seeds/seedlings/plants (ix) Identification of potential SM inducing CEMs by chemical profiling of plant tissue/s (x) Mass multiplication of potential SM inducing CEMs for large scale cultivation with the ultimate aim of increasing microbial heterogeneity. The middle panel represents the key aspects of microbiome engineering with increasing and decreasing complexities in bottom-up and top-down approaches. The various shapes of the microbes indicate different isolates chosen throughout the designing process

The preliminary aspect of microbiome engineering is identifying the core microbiome and host functions provided by individual members and their interaction. This is followed by targeted manipulation of plant microbiome composition to achieve specific phenotypes, in this case, high quantity and quality of secondary metabolites for pharmaceutical and other applications. However, our current knowledge and ability to manipulate microbiomes are limited. We need systematic studies first to identify the interactive impact of plant microbiomes and then identify signal molecules (including volatiles) that trigger metabolite synthesis and microbial modification. This will require the utilization of manipulative work in combination with combined multi-omics techniques like metagenomics, metaproteomics, and metatranscriptomics to determine the functionality of the entire microbial community within a particular niche [225, 226] (Fig. 3).

Identification of signal molecules using sensitive instruments (e.g. GC–MS, HPLC–MS) that trigger plant synthesis and/ or microbial modification can be used to manipulate in situ microbiomes in future. Recently, a four-step cycle was proposed to enable efficient microbiome engineering: design-build-test-learn (DBTL) [227]. This cycle entails creating an initial microbiome design to accomplish a specified engineering goal, building the microbiome, testing its functionality against a set of predetermined metrics to see if the design-build solution(s) produced the designed objective, understanding the outcomes and shortcomings, and utilizing new information for the upcoming DBTL cycles. By following this cycle iteratively, researchers can make step-by-step improvements, enhance the endophyte-host interaction, and achieve targeted enhancement of specific secondary metabolites in the host plant.

### The top-down and bottom-up approach to design microbiomes

Empirical-based attempts to pinpoint the specific functions of individual components in producing community-derived phenotypes are difficult to accomplish due to biology's nonlinearity and the functional complexity present in microbial communities [228]. Therefore, to address the intricacy and to create biotechnological applications, top-down and bottom-up approaches can be adopted to design “customized endomicrobiome”. The top-down design enables the user to select various environmental parameters, which in turn enforces the existing microbiome to remodel itself and modulate to exhibit the desired biological functions [229]. For implementing a top-down approach, microbial resource management is an effective way of predicting the process of manipulating an ecosystem, which requires researchers to intellectualize a system that takes into account different inputs and outputs considering physiological parameters such as pH, redox potential, temperature, humidity etc. and predicts their capabilities to promote or restrict the particular biological process of interest [230]. Whereas, in the bottom-up approach, systems are pieced together to create increasingly sophisticated systems, making the initial systems subsystems of the budding system. When we move from DNA components to microbial ecosystems, this approach requires understanding how the interacting metabolic networks of each microorganism to be incorporated into the microbiome may affect the desired output [223, 227]. This will require a design process involving genomes of key microbiome members [231], reconstructing their metabolic networks [232], and the use of modelling tools [233] and/or network analysis tools [234] to finally come up with a best-fit design. Overall, the bottom-up engineering approach is supported by logical guidance and requires experienced practitioners with the requisite foundation for wise engineering decision-making since all implementation depends on prior knowledge. This approach can be well adapted for systematically enhancing secondary metabolites through endophytic plant partners in controlled conditions. We believe that if the study is precisely designed and constructed to engineer a microbiome, achieving targeted and reliable enhancement of specific secondary metabolites in the host plant is not a very far-off dream.

### Assembling microbiomes for achieving the desired traits in the host plant

New inventive approaches are needed to recruit beneficial microbes that positively impact plant performance. A theoretical network framework was proposed to identify optimal CMs recruited in a microbial network at central positions with associated extra microbes encompassing desired functions for the benefit of the hosts [206]. We thrust upon that utilizing the same approach can potentially enhance secondary plant metabolites production. For example, CEM in medicinal plants can be manipulated/ engineered in two ways: by self-assembly or by synthetic approach. Self-assembled microbiomes can be developed as open consortia using bio-reactors or biostimulators in which the building process actualizes an atmosphere conducive to the growth and desirable activity of the selected microorganisms [227]. Though building up microbiomes by applying axenic microbial cultures results in less complexity, designing synthetic microbiomes is difficult because of significant knowledge gaps about the taxa that are unculturable, uncharacterized and genetically unavailable with potentially substantial roles in agriculture applications. Therefore, to bridge the gap, innovative separation and controlled microbiome assembly methods like single-cell sorting [235] in combination with progressive culturomics [236] and phenotyping [237] can be used to encapture and employ the uncharacterized class of the metabolically diversified community.

Further, a designer assembly can be potentially attained if the latest cell phenotyping and sorting techniques are considered. Raman-activated cell sorting (RACS) is an emerging technology that allows sorting and phenotypic characterization of cells based on their intrinsic biochemical profiles without needing external labelling [238]. Another technique, namely bio-orthogonal non-canonical amino acid tagging (BONCAT), offers an additional approach to analyze microbial anabolic activity *in situ,* which could be coupled with cell sorting to separate active cells from complex samples and further identify them by DNA sequencing [239]. After the cells are individually sorted, culturing through current techniques requires sophisticated setups occupying large spaces. However, advancement in culturomics has introduced microfluidic devices such as microfluidic chips, also known as lab-on-a-chip technology [240–242], which can be further used to create and modify micro-droplets that can facilitate close analysis of axenic cultures with relatively low reagent consumption, elimination of undesired microbial species followed by sequencing, characterization and phenotyping through multi-omics approaches [243]. Moreover, they also offer an additional ability to control microbial communities' density, shape and size [241] with an analysis rate of 100,000 microbiomes/day. Overall, even though no single study has been carried out in the case of medicinal plants in this regard, we firmly believe that information from databanks and associated informatics tools offering the functionality of microbes to the annotated network data can be of great help in designing CEMs for the introduction into medicinal plant endosphere for successfully modulating the plant’s chemistry.

## Conclusion, prospects and key questions

**Fig. 4:**
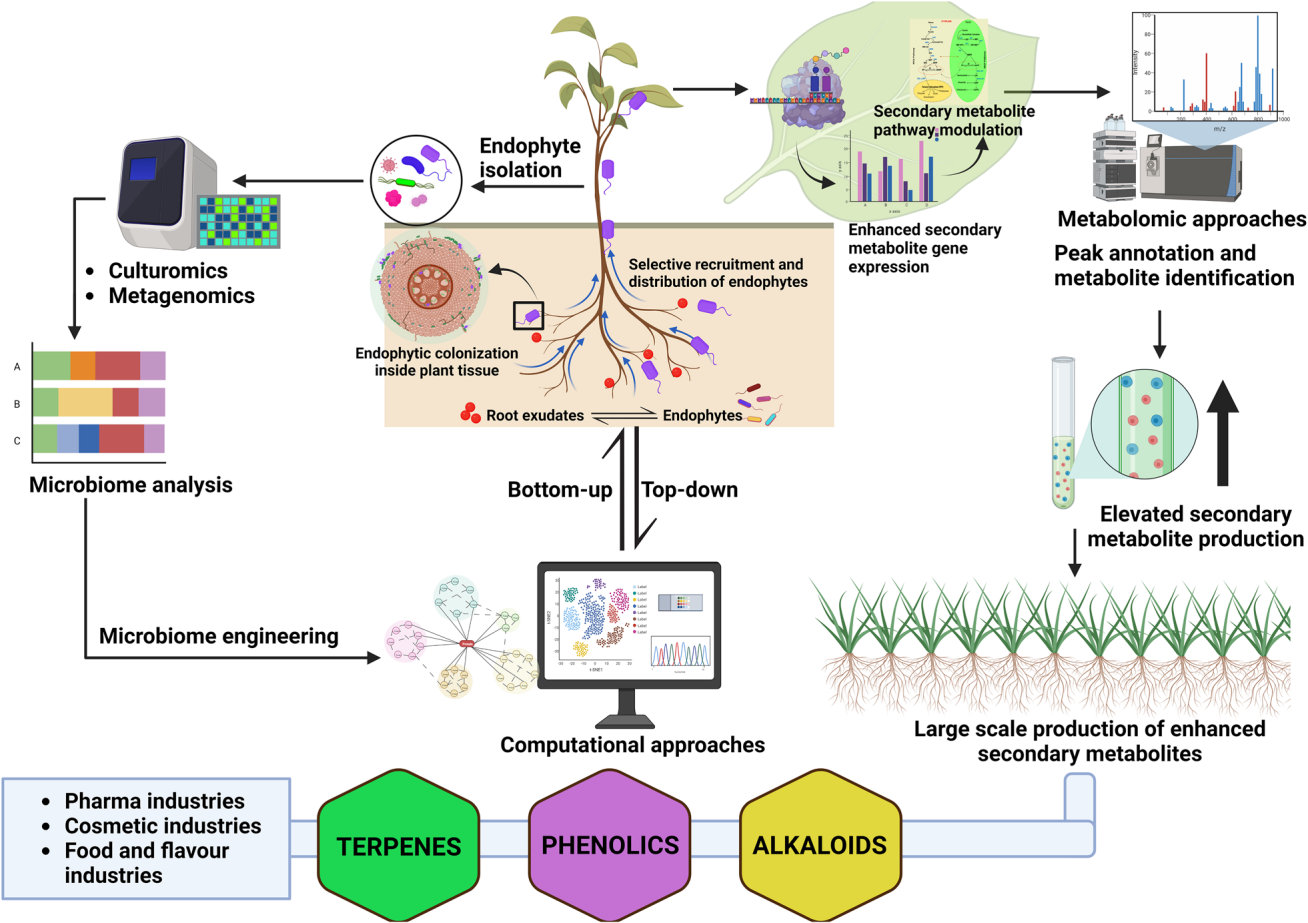
Schematic representation of the complex dynamics of plant-endophyte interactions: Harnessing the power of endomicobiome engineering and mutiomics insights for its practical applications

Endophytes play important roles in manipulating the secondary metabolite biosynthetic pathways with pharmaceutical and other chemical applications. Increasing quality and quantity of secondary metabolite productions are high and demand, and we propose that leveraging the core endomicrobiome of medicinal plants can provide effective solutions. However, owing to the complexity and multitude of interactions between microbiome-plant and the large diversity of plant microbes that remains poorly described, the precise engineering of CEMs for producing secondary metabolites will need concerted planning and collective efforts. The emerging tools of omics combined with the application of microfluidics, synthetic biology, genome editing, machine learning and data designing provide an opportunity to carry out large-scale endomicrobiome and plant/microbial metabolite screening. The success of such approaches will require transdisciplinary approaches involving active collaborations among agriculturists, chemists, engineers, microbiologists, plant molecular biologists/biochemists, and big data analysts. Based on the recent advancements in research tools and techniques, there is an opportunity to transform the production of secondary metabolites in medicinal plants by harnessing core microbiomes and plant-microbiome interactions through sustained resourcing and systems-based approaches (Fig. 4). In addition, we believe that by addressing some important key questions given below, the lacunas in the endophyte plant secondary metabolite enhancement can be bridged.

1. Are there any specific core endophytic species or strains consistently associated with all the medicinal plants in general that hold promise for discovering new drugs or therapeutic agents?
2. Considering their vast diversity, how can effective endophytes be identified for specific metabolite enhancement?
3. How do endophytes establish and maintain their interactions with medicinal plants, which are rich reservoirs of antimicrobial compounds?
4. Have the medicinal plants and their associated endophytes coevolved over time, and can investigating the drivers of host specificity and the ecological factors provide insights into the evolution of these interactions?
5. What factors contribute to species-specific and variable abilities of endophytes to enhance secondary metabolites?
6. How do host-endophyte interactions and environmental conditions influence the efficacy and reliability of endophyte applications?
7. Can understanding the functional variation among different endophyte taxa and their effects on host plants help harness their potential for medicinal plant cultivation?
8. How can we better understand the functional consequences of shifts in endophyte diversity in response to plant metabolites?
9. How can advanced genomic tools and approaches effectively address the gaps and challenges in studying endophytes and plant secondary metabolism?

### Supplementary Information

**Additional file 1: Table S1.** List of beneficial endophytic microbes associated with various medicinal plants and their plant growth promoting (PGP) and antimicrobial properties. **Table S2.** List of endophytes enhancing pharmaceutically important secondary metabolites in medicinal plants.

## Data Availability

Not applicable.

## Abbreviations

CM: Core microbiome
CEM: Core endomicrobiome
