# Correction: Plants and endophytes interaction: a “secret wedlock” for sustainable biosynthesis of pharmaceutically important secondary metabolites

**DOI:** 10.1186/s12934-023-02281-1

**Published:** 2024-01-02

**Authors:** Poonam Kumari, Nikky Deepa, Prabodh Kumar Trivedi, Brajesh K. Singh, Vaibhav Srivastava, Akanksha Singh

**Affiliations:** 1 Division of Crop Production and Protection, Central Institute of Medicinal and Aromatic Plants, Lucknow, 226015 India; 2 Division of Plant Biotechnology, Central Institute of Medicinal and Aromatic Plants, Lucknow, 226015 India; 3 Hawkesbury Institute for the Environment, Western Sydney University, Penrith, NSW 2753 Australia; 4 Global Centre for Land-Based Innovation, Western Sydney University, Penrith, NSW 2751 Australia; 5 Division of Glycoscience, Department of Chemistry, School of Engineering Sciences in Chemistry, Biotechnology and Health, KTH Royal Institute of Technology, AlbaNova University Center, 106 91, Stockholm, Sweden; 6 Academy of Scientific and Innovative Research (AcSIR), Ghaziabad, India

**Correction: Microbial Cell Factories (2023) 22:226**

10.1186/s12934-023-02234-8

In this article [1] the affiliation details for the corresponding author, Vaibhav Srivastava were incorrectly given as '5,6' but should have been '5'. The corrected affiliation details of the author should be “Division of Glycoscience, Department of Chemistry, School of Engineering Sciences in Chemistry, Biotechnology, and Health, KTH Royal Institute of Technology, AlbaNova University Center, 106 91 Stockholm, Sweden”. The original article has been corrected.
