# Supplementary material for: Plants and endophytes interaction: a “secret wedlock” for sustainable biosynthesis of pharmaceutically important secondary metabolites
Source: Microb Cell Fact. 2023 Nov 4;22:226. doi: 10.1186/s12934-023-02234-8 (PMC10625306; doi:10.1186/s12934-023-02234-8)
Supplement: Supplementary file 1 — Additional file 1: Table S1. List of beneficial endophytic microbes associated with various medicinal plants and their plant growth promoting (PGP) and antimicrobial properties. Table S2. List of endophytes enhancing pharmaceutically important secondary metabolites in medicinal plants. [file 12934_2023_2234_MOESM1_ESM.pdf]

Additional File 1

Table S1. List of beneficial endophytic microbes associated with various medicinal plants and their plant growth promoting (PGP) and antimicrobial properties

S. No.	Endophytes	Host Plant	Plant Part	PGP/ Antimicrobial Properties	References
1.	*Bacillus cereus* and *B.subtilis*	*Teucrium polium* L.	Leaves	Production of indole-3- acetic acid (IAA), ammonia and phosphate solubilization trait	[50]
2.	*B. subtilis, Agrobacterium tumefaciens, Bacillus* sp., *Pseudomonas putida,* and *Pseudomonas* sp.	*Casia tora*	Root	Synthesis of IAA, ammonia, siderophores, HCN and solubilization of phosphate	[51]
3.	*Paenibacillus* sp.	*Tridax procumbens*	Root	Production of secondary metabolites, IAA, siderophores, ACC deaminase, biosurfactant and phosphate solubilization	[52]
4.	*B. cereus* (ECL1)*, B. thuringiensis* (ECL2), *Bacillus* sp. (ECL3), *B. pumilis* (ECL4), *P. putida* (ECL5), and *Clavibacter michiganensis* (ECL6)	*Curcuma longa* L.	Rhizome	Production of IAA, siderophore and phosphate solubilization	[53, 54]
	Achromobacter xiloxidans,				

	Alcaligenes sp., *Bacillus pumilus*				
5.	*Enterobacter* sp., *Rahnella* sp., *Rhodanobacter* sp., *Pseudomonas* sp., *Stenotrophomonas* sp., *Xanthomonas* sp. and *Phyllobacterium* sp.	*Ipomoea batatas* L.	Stem	Production of IAA, nitrogen fixation and stress tolerance	[55]
6.	*B. subtilis* and *B. thuringiensis*	*Glycine max* L.	Seed	Increase in nodule number per plant, increased soybean weight	[56]
7.	*Pseudomonas* sp.	*Zingiber officinale*	Rhizome	IAA, ACC deaminase and siderophore production	[57]
8.	*Achromobacter xiloxidans*; *Alcaligenes* sp., *B. pumilus*	*Helianthus annus* L.	Root	Positive catalase and oxidase activities, phosphate solubilization	[58]
9.	*Bacillus* sp., *Sphingopyxis* sp.	*Fragaria ananassa*	Meristematic tissue	Indole acetic acid production, solubilization of inorganic phosphate	[59]
10.	*B. subtilis* OS-11	*Ocimum sanctum*	Leaves	Increase in the content of essential oil, enhancement of plant growth	[60]
11.	*Bacillus* sp. and *Enterobacter* sp.	*Phoenix dactylifera* L.	Seed	ACC deaminase, IAA production, ammonia production, ferric iron chelation, solubilization of potassium, phosphorus and zinc	[61]
12.	*Psuedomonas resinovorans, Paenibacillus polymaxa*, and *Acenitobacter calcoaceticus*	*Gynura procumbens*	Leaves	Production of cytokinin compounds	[62]
13.	*Pseudomonas* sp. Ph6- gfp	*Trifolium pratense* L.	Root	Degradation of phenanthrene, a toxic	

				metabolite	[63]
14.	*Acinetobacter* sp. ALEB16	*Atractylodes lancea*	Leaves	Accumulation of plant volatile oils and induction in abscisic acid and salicylic acid production	[64]
15.	*Bacillus thuringiensis* GDB-1	*Pinus sylvestris*	Root	ACC deaminase activity, IAA and siderophore production along with phosphate solubilization trait	[65]
16.	*Bacillus subtilis* HYT- 12-1	*Lycopersicum esculentum*	Seed	IAA production; phosphate solubilization; siderophores production; nitrogen fixation and ACC deaminase activity	[66]
17.	*Achromobacter xylosoxidans* strain AUM54	*Catharanthus roseus*	Root	ACC deaminase activity, increased plant ethylene levels and increased antioxidative enzyme	[67]
18.	*Bacillus megaterium, Bacillus pumilus, Bacillus licheniformis, Micrococcus luteus, Paenibacillus* sp., *Pseudomonas* sp., and *Acinetobacter calcoaceticus*	*Plectranthus tenuiflorus*	Root, stem, and leaves	Production of extracellular enzymes	[68]
19.	*Bacillus amyloliquefaciens* ES-2	*Scutellaria baicalensis*	Root	Production of secondary metabolites with broad-spectrum antibacterial and antifungal activities	[69]
20.	*Serratia nematodiphila* LRE07, *Enterobacter aerogenes* LRE17, *Enterobacter* sp. LSE04 and	*Solanum nigrum* L.	Root, stem, and leaves	ACC deaminase, indole acetic acid, siderophore and phosphate solubilizing activity	[70]

	Acinetobacter sp. LSE06				
21.	*Acinetobacter* sp., *Agrobacterium* sp., *Bacillus* sp., *Brevibacillus* sp., *Burkholderia* sp., *Curtobacterium* sp., *Erwinia* sp., *Lactococcus* sp., *Pantoea* sp., and *Pseudomonas* sp.	*Eucalyptus* sp.	Stem	Production of new metabolites and enzymes and control of plant diseases	[71]
22.	*Arthrobacter* sp. SMR3, *B. subtilis* SMR15	*Papaver somniferous*	Root, leaves, capsule and seeds	Benzylisoquinoline alkaloid (BIA) biosynthesis, IAA production, ACC deaminase production	[72]
23.	*Alcaligenes faecalis* subsp. faecalis str. S8	*Withania somnifera*	Stem and fruit	Production of IAA and phosphate solubilization	[73]
24.	*B. subtilis* LK14	*Moringa peregrine*	Bark	Phosphate solubilization, ACC deaminase and acid phosphatase activity	[74]
25.	*Paenibacillus validus, Lysinibacillus fusiformis, B. licheniformis, Pseudomonas putida, Microbacterium oleivorans,* and *Serratia plymutica*	*Citrus sinensis*	Root	Phosphate solubilization, siderophore production, nitrogen fixation, IAA synthesis, production of antibiotic and lytic enzymes (chitinase), induction of systemic resistance through salicylic acid production	[75]
26.	*Bacillus* sp., *Pseudomonas* sp.	*Bauhinia purpurea*	Stem, leaves and flower	Appreciable antioxidant and antimicrobial activity	[76]
27.	*Acinetobacter* sp. ACMS25 and *Bacillus* sp. PVMX4	*Phyllanthus amarus*	Root	Phosphate solubilization, IAA production, siderophore production, ACC deaminase activity and	[77]

				production of hydrolytic enzymes like cellulose, protease, pectinase	
28.	*Azotobacter* sp., *Azotobacter vinelandii, Azotobacter chroococcum*	*Vigna radiata*	Root and leaves tissues	IAA production, phosphate solubilizing activities	[78]
29.	*Bacillus* sp., *Enterobacter* sp. and *Sporosarcina aquimarina*	*Avicennia marina*	Pneumato-phores	Production of siderophore, phosphate solubilization and IAA production	[79]
30.	*Pseudomonas fluorescens* G10 and *Microbacterium* sp. G16	*Brassica napus*	Root	Production of IAA, siderophores and ACC deaminase resulting in increased biomass production	[80]
31.	*Burkholderia phytofirmans*	*Allium Cepa*	Root	ACC deaminase activity	[81]
32.	*Acetobacter diazotrophicus*	*Saccharum officinarum*	Stem	Nitrogen fixation ability	[82]
33.	*Bacillus subtilis*	*Morus alba*	Root, stem and leaves	Reduced bacterial wilt	[83]
34.	*Burkholderia cepacia, Rhizobium* sp., *Klebsiella* sp., *Gluconacetobacter diazotrophicus, Herbaspirillum seropedicae, Pantoea agglomerans, Azospirillum* sp., *Bacillus* sp., and *Enterobacter* sp.	*Zea mays*	-	Growth enhancement	[84]
35.	*Bacillus megaterium*	*Zea mays*	Root, stem and leaves	-	[85]
36.	*Burkholderia vietnamiensis*	*Saccharum officinarum*	Root	Growth enhancement and increased	[86]

				yield	
37.	*Pseudomonas* sp.	*Populus* sp.	Root, stem and leaves	Ability to degrade 2,4-dichlorophenoxyacetic acid (2,4-D)	[87]
38.	*Gluconacetobacter diazotrophicus, Herbaspirillum seropedicae, Herbaspirillum rubrisubalbicans, Azospirillum amazonense, Burkholderia tropica*	*Saccharum officinarum*	Root	-	[88]
39.	*Gluconacetobacter diazotrophicus, H. seropedicae, Herbaspirillum rubrisubalbicans, A. amazonense* and *Burkholderia* sp.	*Saccharum officinarum*	Root and shoot	Growth enhancement and increased nitrogen content	[89]
40.	*Klebsiella aerogenes, B. subtilis*	*Bacopa monnieri*		Enhancement in plant biomass and Bacoside A content	[19]
41.	*Sphingomonas* sp., *Pseudomonas* sp., *Lasiodiplodia theobromae, Penicillium digitatum, Aspergillus awamori, Trichoderma harzianum, Aspergillus flavus, Cladosporium herbarum*, *A. niger*, *Monascus* sp., *Penicillium citrinum*,	*Solanum nigrum* L.	Stem, leaves and fruit	-	[90, 91, 92, 93]

	and *Penicillium chrysogenum*				
43.	*Aspergillus* sp., *Cladosporium* sp.	*Calotropis procera*	Root, stem and leaves	Antimicrobial and antioxidant activities	[94]
44.	*Alternaria porri, Aspergillus flavipes, Fusarium oxysporum, Nigrospora sphaerica, A. niger, Curvularia lunata, Colletotrichum falcatum, Phomopsis archeri, Pestalotiopsis sydowiana, Phoma exigua,* and *Leptospharulina chartarum*	*Calotropis. Gigantean*	Stem, leaves and flower	-	[95]
46.	*Delftia tsuruhatensis , Pseudomonas frederiksbergensis, Agrococcus lahaulensis, Arthrobacter, B. siamensis, B. tequilensis, B. zhangzhouensis, Chryseobacterium cucumeris, Kocuria polaris* and *Sphingomonas genera*	*Scrophularia striata*	Root, stem and leaves	Antibacterial activity against plant pathogens	[96]
47.	*Pseudomonas* sp. and *Bacillus* sp.	*Aloe barbadensis*	Root, stem and leaves	-	[97]
48.	*P. aeruginosa, P. putida, B. megaterium, Arthrobacter* sp., *Micrococcus* sp.,	*Piper nigrum* L.	Root and stem	Antagonistic activity against *Phytophthora capsici*	[98]

	Curtobacterium sp.				
49.	*Erwinia* sp., *Pseudomonas* sp., *B. megaterium*, *B. chosinensis*, and *Microbacterium trichothecenolyticum*	*Medicago sativa* L.	Root	-	[99, 100]
50.	*B. siamensis*	*Coriandrum sativum*	-	Increased root length, shoot length, and dry weight	[101]
51.	*Micrococcus luteus* and *Lysinibacillus fusiformis*	*Panax quinquefolius*	Stem	Enhanced seedling biomass	[102]
52.	*Bacillus* and *Paenibacillus* sp.	*Curcuma longa*	Rhizome	Increased root length, shoot length, and root number	[103]
53.	*Bacillus* sp.	*Curcuma longa*	Rhizome	Induced host disease resistance	[104]
54.	*Pseudomonas aeruginosa*	*Anredera cordifolia*	Leaves	Production of proteases, amylase, esterase, and cellulose enzymes	[105]
55.	*B. subtilis*	*Pseudobrickellia brasiliensis*	Stem and leaves	Protease and esterase activity	[106]
57.	*P. aeruginosa*	*Achyranthes aspera*	Leaves	Improved plant biomass and antioxidant activity	[107]

Table S2. List of endophytes enhancing pharmaceutically important secondary metabolites in medicinal plants

S. No.	Endophyte	Host plant	Secondary plant metabolite/s	Reference
1.	*Colletotrichum gloeosporioides*	*Centella asiatica*	Asiaticoside	[154]
2.	*Fusarium* sp.	*Dracaena cochinchinensis*	Red resin	[155]
3.	*Piriformospora indica*	*Bacopa monnieri*	Bacoside	[156]
4.	*Phomopsis* sp.	*Euphorbia pekinensis*	Terpenoid	[157]
5.	*Piriformospora indica*	*Spilanthes calva*	Spilanthol	[158]
6.	*Sebacina vermifera*	*Linum album*	Podophyllotoxins	[159]
7.	*Fusarium mairei*	*Taxus chinensis*	Paclitaxel	[160]
8.	*Fusarium mairei*	*Taxus cuspidate*	Paclitaxel	[161]
9.	Endophytic fungus	*Catharanthus roseus*	Alkaloid	[162]

10.	*Piriformospora indica*	*Curcuma longa*	Curcumin and volatile oil	[163]
11.	*Ramularia* sp.	*Rumex gmelini Turcz*	Emodin	[164]
12.	*Fusarium* sp.	*Rumex gmelini Turcz*	Musizin, chrysophanol and physcion	[164]
13.	*Aspergillus oryzae*	*Rumex gmelini Turcz*	Resveratrol	[164]
14.	*Trimmatostroma* sp.	*Rhodiola crenulata*	Salidroside and tyrosol	[165]
15.	*Rhizophagus intraradices*	*Hypericum perforatum* L.	Hypericin and pseudohypericin	[166]
16.	*Piriformospora indica*	*Withania somnifera*	Withaferin A	[167]
17.	*CATDLF5 (Curvularia sp.) and CATDLF6 (Choanephora*	*Catharanthus roseus*	Serpentine	[17]

	infundibulifera)			
18.	*CATDS5 (Pseudomonas sp.)*	*Catharanthus roseus*	Ajmalicine	[17]
19.	*Burkholderia* sp.	*Artemisia annua*	Artemisinin	[168]
20.	*Gilmaniella* sp.	*Atractylodes lancea*	Ethylene	[169]
21.	*Chaetomium globosum & Colletotrichum gloeosporioides*	*Anoectochilus roxburghii*	kinsenoside	[170]
22.	*Pseudomonas fluorescens*	*Atractylodes lancea*	Essential oil	[171]
23.	*Paenibacillus polymyxa*	*Panax ginseng*	Ginsenoside	[152]

24.	*Bacillus subtilis*	*Ligusticum chuanxiong*	ligustrazine	[172]
25.	*Pseudonocardia* sp.	*Artemisia annua*	Artemisinin	[173]
26.	*Burkholderia* sp.	*Panax ginseng*	Ginsenoside Rg3	[129]
27.	*Ilyonectria liriodendra*	*Houttuynia cordata*	Chlorogenic acid, quercitrin α-pinene, β-pinene, camphene, β-myrcene, limonene, decanal, bornyl acetate, β-caryophyllene, 2-undecanone	[12]
28.	Arbuscular mycorrhiza fungi (AMF) and *Trichoderma konigii*	*Ocimum basilicum*	Ferulic acid, chicoric acid, and quercetin rutinoside	[174]
29.	*Mucor fragilis*	*Salvia miltiorrhiza*	Diterpenoides and phenolic acids	[11]

30.	*Staphylococcus sciuri* and *Micrococcus* sp.	*Catharanthus roseus*	Terpenoid indole alkaloids like vindoline, serpentine and ajmalicine	[175]
31.	Endophytic bacterial community	*Putterlickia retrospinosa* and *P. verrucosa*	Maytansine	[176]
32.	*Azotobacter chroococcum* CL13	*Curcuma longa*	Phenolic compounds, sesquiterpenoids and curcuminoids	[177]
33.	*Stenotrophomonas maltophilia* (N5-18)	*Pappaver sominiferum*	Morphine and total alkaloid	[178]
34.	*Gilmaniella* sp. AL12	*Atractylodes lancea*	Volatile content	[179]
35.	*Bradyrhizobium* sp.	*Crotalaria*	Antiherbivore, nematicide	[180]

36.	*Pantoea dispersa* and *Penicillium polonicum*	*Aquilaria malaccensis*	Agarospirol	[149]
37.	*Paenibacillus* sp.	*Populus* sp.	Threitol and asparagine	[181]
38.	*Enterobacter ludwigii*	*Vitis vinifera*	Vanillic acid	[182]
39.	Bacterial endophytes	*Vetiveria zizanioides*	Essential oil biogenesis	[151]
40.	*Pseudomonas aeruginosa* and *Pseudomonas pseudoalcaligenes*	*Hyptis suaveolens*	Essential oils	[183]
41.	*Bacillus amyloliquefaciens, Pseudomonas fuorescen, Alternaria alternata, Sclerotium rolfsii Aspergillus terreus, Penicillium oxalicum, Sarocladium kiliense*	*Withania somnifera*	Withanolide modulation	[184]

42.	*Bacillus* and *Enterobacter* sp.	*Thymus vulgaris*	Bioactive metabolites (benzene, 1,3-dimethyl-, p-xylene, dibutyl phthalate, bis (2-ethylhexyl) phthalate, and tetracosane)	[185]
43.	*Macrophomina pseudophaseolina* (SF2)	*Andrographis paniculata*	Andrographolide content	[186]
44.	*Fusarium redolens* (RF1), *Phialemoniopsis cornearis* (SF1), and *Macrophomina pseudophaseolina* (SF2)	*Coleus forskohlii*	Forskolin content	[147, 187]
45.	*Piriformospora indica*	*Coleus forskohlii*	p-cymene	[188]
46.	*Glomerella magna,*	*Lycoris radiata*	Alkaloides (narciclasine,	[39]

	Phyllosticta capitalensis, Stagonosporopsis cucurbitacearum, Stagonosporopsis ligulicola, Phyllosticta ophiopogonis and *Colletotrichum gloeosporioides*		lycorine, galanthamine and tazettine)	
47.	*Pseudomonas fluorescens*	*Atractylodes lancea*	Sesquiterpenoid	[189]
48.	*Aspergillus terreus, Penicillium oxalicum, Sarocladium kiliense* (endophytes) and, *Trichoderma viride* (biocontrol agent)	*Withania somnifera*	Withaferin A	[190]
49.	*Alcaligenes faecalis*	*Coleus forskohlii*	Forskolin	[186]

50.	*Chitinophaga* sp., *Allorhizobium* sp., *Duganella* sp., and *Micromonospora* sp.	*Alkanna tinctoria*	Alkannin and shikonin	[16]
51.	*Piriformospora indica*	*Centella asiatica*	Asiaticosides	[191]
52.	*Mycena* sp.	*Anoectochilus formosanus*	Flavonoid and kinsenoside	[153]
53.	*Bacillus subtilis* and *Klebsiella aerogenes*	*Bacopa monnieri*	Bacoside	[19]
54.	*Paenibacillus* sp.	*Bacopa monnieri*	Bacoside A	[18]
55.	Bacterial endophytes	*Echinacea purpurea*	Alkamides, volatile organic compounds (VOCs) and phenylpropanoid compounds	[192, 193]
